# The Environmental and Social Influences of HIV/AIDS in Sub-Saharan Africa: A Focus on Rural Communities

**DOI:** 10.3390/ijerph8072967

**Published:** 2011-07-19

**Authors:** Christine U. Oramasionwu, Kelly R. Daniels, Matthew J. Labreche, Christopher R. Frei

**Affiliations:** The University of Texas at Austin, College of Pharmacy, and The University of Texas Health Science Center San Antonio, Department of Medicine, Pharmacotherapy Education and Research Center, 7703 Floyd Curl Drive, MC-6220, San Antonio, TX, 78229-3900, USA; E-Mails: kdaniels@mail.utexas.edu (K.R.D.); labreche.matthew@gmail.com (M.J.L.); freic@uthscsa.edu (C.R.F.)

**Keywords:** HIV/AIDS, environment, sub-Saharan Africa, rural

## Abstract

The Human Immunodeficiency Virus/Acquired Immunodeficiency Syndrome (HIV/AIDS) pandemic has caused far-reaching effects in sub-Saharan Africa. The pandemic has effectively diminished the workforce, increased poverty rates, reduced agricultural productivity, and transformed the structure of many rural households. HIV/AIDS further strains the already fragile relationship between livelihood and the natural and social environments of these regions. Therefore, the objective of this review is to characterize the impact of HIV/AIDS on the environment and the social infrastructure of rural sub-Saharan Africa. There are many aspects of rural life that contribute to disease transmission of HIV/AIDS and that pose unique challenges to the population dynamics in sub-Saharan Africa. Widespread AIDS-related mortality has caused a decrease in population growth for many African countries. In turn, these alterations in population dynamics have resulted in a decrease in the percentage of prime-age working adults, as well as a gender disparity, whereby, females carry a growing burden of household responsibilities. There is a rising proportion of older adults, often females, who assume the role of provider and caretaker for other dependent family members. These changing dynamics have caused many to exploit their natural surroundings, adopting less sustainable land use practices and utilizing protected resources as a primary means of generating revenue.

## 1. Introduction

The Human Immunodeficiency Virus/Acquired Immunodeficiency Syndrome (HIV/AIDS) pandemic has caused far-reaching effects in resource-limited settings. The continent of Africa has been particularly hard hit. It is estimated that two-thirds of the 33 million people living with HIV/AIDS worldwide, reside in this region, with a rising majority in rural areas [1]. Based on data from 2008, an estimated 71% of all new infections, and 72% of AIDS-related deaths, occurred in sub-Saharan Africa [1]. In response to these dismal statistics, the international community has responded by raising awareness of the medical needs of this region. These scale-up efforts have been quite successful as millions of Africans are gaining access to life-saving antiretroviral therapies [2,3].

While much attention has focused on the medical aspects of HIV/AIDS, other issues beyond the medical sector have emerged. HIV/AIDS further constrains the already fragile relationship between livelihood and the environment, as thoroughly described by Niehof, Rugalema, and Gillespie [4]. Rural livelihood depends on several factors, including, natural resources, technology, knowledge, health, and access to education; many of which have been influenced by the pandemic. For instance, HIV/AIDS has greatly altered the population dynamics of this region by reducing the agricultural workforce. To compensate, many farmers have adopted less labor-intensive, unsustainable land use practices in an attempt to increase crop yield and generate revenue, or have left crops unseeded as a result of the diminished availability of laborers [5]. Such issues are especially problematic in rural communities where many inhabitants depend on the natural environment as a source of income and as a means of preserving traditional and cultural practices [6].

The juxtaposition of these factors evokes the question: what is the environmental impact of HIV/AIDS on the rural communities of sub-Saharan Africa? This review discusses the attributes of rural life that influence HIV/AIDS in sub-Saharan Africa, describes the fluctuations in population dynamics as a result of the pandemic, and characterizes the variations in the management and use of the local environment. It also highlights recommendations to lessen and reverse the effects of HIV/AIDS on the natural environment in this region.

## 2. Literature Assessment

Searches of the MEDLINE and GreenFILE databases were conducted to identify pertinent articles. The searches were restricted to articles published in peer-reviewed journals in the English language. The terms *“HIV”*, *“Africa”*, *“rural”* and *“environment”* were used alone and in combination to yield the maximum number of articles within each database. Of the 147 publications identified, 13 were included for meeting all of the following inclusion criteria: pertained to the environment, described original research findings, involved patients with HIV/AIDS, location in sub-Saharan Africa, and focused on issues pertaining to rural community life. Publications failing to meet the above criteria were excluded. The citations of relevant articles were also reviewed to yield an additional 18 articles. The articles are classified by focus area in Table 1.

## 3. HIV/AIDS in Rural Sub-Saharan Africa

### 3.1. The Rural Dimension of HIV/AIDS

The various HIV/AIDS epidemics across sub-Saharan Africa are diverse in terms of rate of spread and characteristic variations from region to region [34]. The prevalence of disease tends to be greater in urban areas as compared to rural areas, with the urban-rural ratio estimated at 1.7:1.0 [35]. However, there are also unique factors to rural life that contribute to disease transmission. For example, HIV/AIDS clinics and other related services are not as prevalent in villages and surrounding vicinities as they are in densely-populated cities; thus, limiting the amount of care as well as disease and treatment-related knowledge that reaches rural dwellers [36]. This disparity in equitable access to care between rural and urban areas is steadily gaining awareness, but requires more progress to meet the needs of these areas [37]. Many of these communities already face escalating food costs and decreased labor wages due to political instability and economic strife, which has given rise to food insecurity and malnutrition [5,27,38]. Concurrently, the pandemic diminishes the workforce, increases poverty rates, reduces agricultural productivity, and transforms the structure of many rural households [9,10,19]. This complex interaction does not always yield negative results, it merely reinforces the importance of recognizing the diversity of impacts from this disease [11,30,34].

### 3.2. Rural Migration

The migratory tendencies of infected individuals in these areas further complicate rural life for people with HIV/AIDS. Previous studies of migration patterns have noted that HIV-infected individuals often return to their home village in order to be closer to relatives that can provide and care for them while they are ill [14,15]. This process, in turn, may increase the risk of transmission to others in home villages. Migration patterns among travelling laborers also impacts the spread of HIV/AIDS in rural communities, particularly by young male workers. Researchers were able to describe a pattern in which workers would leave their rural homes in search of work in the city by modeling the movement of workers in rural South Africa [16]. Once in the city, there was a tendency for workers to engage in high-risk sexual behavior, thus, increasing the risk for disease acquisition to others [16]. Once infected, their return to their rural home increased the risk of transmission to their partners and to other members of their village [16]. These multiple avenues for the spread and introduction of HIV/AIDS into the rural community may serve as areas for effective intervention.

## 4. Changes in Population Dynamics

### 4.1. Population Growth

**Figure 1:**
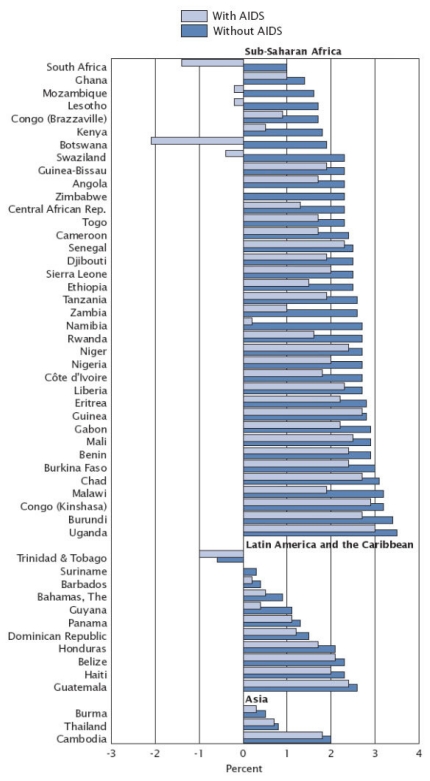
Population growth rates with and without AIDS for selected countries [39].

One of the most notable consequences of the HIV/AIDS pandemic has been the reduction in population growth as a result of AIDS-related mortality. A report produced by the U.S. Census Bureau in 2004 indicated that many sub-Saharan African countries are projected to experience a decline in life expectancy [39]. This report forecasted that by 2010, life expectancy would have dropped to nearly 30 years of age for some South African countries, levels that were last observed at the start of the 20th century [39]. At greatest risk for this reversion are countries with a high prevalence of HIV/AIDS. For instance, Botswana, Lesotho, Mozambique, South Africa, and Swaziland each have a HIV seroprevalence greater than 20% [39]. Based on modeling, these countries were all expected to experience negative population growth attributable to AIDS by 2010; these projected growth rates (with and without AIDS) are illustrated in Figure 1 [39]. These dramatic changes in population growth impact rural livelihoods. Analyzing the effects of the growth and reduction of certain subgroups within the population at large reveals the array of complexities and challenges that arise from declines in population growth, as will be discussed in subsequent sections of this review.

### 4.2. The Age Dynamic

Increased mortality due to AIDS, will not only reduce the overall population, but will also distort the age structure of many of these countries [39]. Such demographic information is best depicted using a population pyramid. The population pyramid is a graphical representation of the different age groups for a given country and typically resembles a ‘pyramid’ shape; this shape denotes population growth. One way to discern the impact of AIDS-related mortality is to compare population pyramids for different countries with varying disease burden.

**Figure 2:**
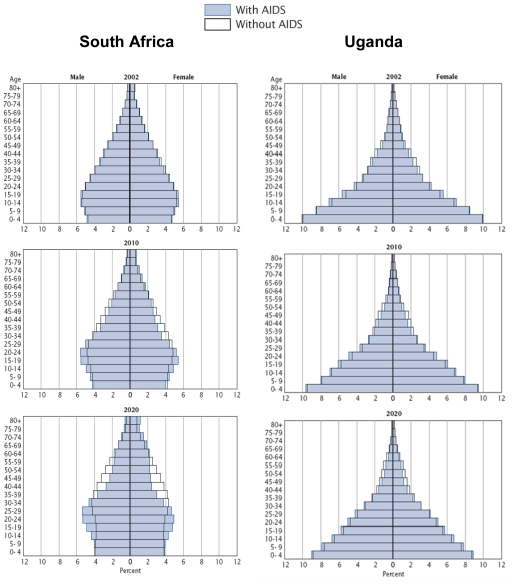
Population by age and gender with and without AIDS for South Africa and Uganda: 2002, 2010, and 2020 (adapted from the U.S. Bureau of the Census) [39].

The deleterious impact of disease on population growth is a mounting challenge for countries with high seroprevalence. First, consider that the estimated seroprevalence in 2001 was 20% in South Africa and 5% in Uganda [39]. Because of this, South Africa will progressively veer away from the traditional pyramid shape with a predominant loss of the younger and adult populations, indicative of negative population growth, as illustrated in Figure 2. In contrast, very little changes in the population are expected to occur in Uganda.

The age distortion of the population influences the rural family unit. In South Africa, the percentage of the adult population is decreasing, yet the percentage of the elderly population is increasing. This is hypothesized to be a result of decreasing fertility rates among women, low levels of life expectancy among children born with HIV/AIDS, and adult AIDS mortality [40]. Suppression of urban growth rates in South Africa, as well as other parts of sub-Saharan Africa, has disproportionately affected young and middle-aged adults; however, the impact of HIV/AIDS may become more widely distributed across age groups over time [40].

Elders often resume primary responsibility for the household following the death of their adult children, as demonstrated by an interview of women sampled from South African villages by Schatz *et al*. [17]. The authors described that many of these older women have access to government pensions to support themselves; however, in the absence of their income-producing adult children, these funds are often insufficient for an entire household [17]. Older females are left to cope with these monetary constraints and food insecurities while still caring for children and sick family members [17]. As the family dynamic and economic outlets shift, these women have little time to adopt sustainable use of the available natural resources.

### 4.3. The Gender Dynamic

Approximately 60% of individuals living with HIV/AIDS in sub-Saharan Africa are female [1]. In contrast, females only accounted for 25% of all HIV/AIDS diagnoses among adults and adolescents in the United States in 2008 [41]. Women are at higher risk of acquiring HIV through heterosexual transmission due to physiology; however, other social, cultural, legal, and economic factors may play a role in producing the disproportionately higher rates in women living in sub-Saharan Africa [42]. Some African cultures allow for the subordination of females, whereby, women may lack economic independence, assets, and the ability to take charge of their sexual existence. Dunkle *et al*. demonstrated that women with violent or controlling male partners were at increased risk for HIV infection [20]. The effect of AIDS-related mortality on gender imbalance can also be seen in the two population pyramids in Figure 2. By 2020, the population of South Africa is projected to experience a gender imbalance, wherein males aged 15–44 years will likely outnumber their female counterparts [39]. These gender imbalances are aggravated by HIV/AIDS and continue to present a formidable challenge for females.

In addition to the numerous farming responsibilities, women must also face property inheritance issues. Female control of agricultural households typically transpires following the death of a male spouse, often from AIDS. This raises the concern of land tenure and ownership. Land in sub-Saharan Africa is traditionally inherited through a patriarchal lineage; thus, widowed women are vulnerable to losing land rights upon their spouse’s death [18,28]. Kanyamurwa *et al*. conducted a study of farming households in Uganda to assess the gender influence on the livelihood of homes affected by AIDS and those unaffected by the disease [21]. The investigators concluded that female-headed-households were more affected by AIDS than male-headed-households; women were more prone to losing ownership of their land and livestock [21]. In contrast to the findings of Kanyamurwa *et al*., Aliber *et al*. did not detect a link between HIV/AIDS mortality and land security for females, yet, the authors still concluded that HIV/AIDS does further complicate the issue of land tenure for females [28]. The concern for land loss amidst mounting caretaker duties for females can have direct environmental consequences either as a result of little time to devote to conservative farming techniques, or the transference of the land to owners that have no working knowledge of proper land management [28].

## 5. Management of the Natural Environment

### 5.1. Unsustainable Use of Natural Resources

Many communities have attempted to cope with the HIV/AIDS pandemic by increasing their dependence on the physical environment as a way to maintain their livelihood. Unfortunately, this is often done in a manner that is unsustainable. The agricultural needs at present may compromise future needs; therefore, environmental health, economic viability, and overall quality of life for farmers and society may decline. The Food and Agriculture Organization of the United Nations (FAO) has recognized and characterized the link between HIV/AIDS and the natural environment. The FAO has cited rural Africa as especially influenced by this link, given the reliance of these communities on agricultural activities [43]. An example of the relationship between HIV/AIDS and the natural environment is that forest reserves are being depleted at an alarming rate to generate firewood for households caring for the sick and to manufacture coffins to bury the deceased [44]. Misiko *et al*. sought to describe the trend in the abandonment of proper soil fertility management in Kenyan households afflicted by AIDS [30]. The authors found that families consisted primarily of dependents (children less than ten years of age) and only had a maximum of two members that were involved in subsistence farming [30]. Given the farmland labor constraints, many households had abandoned more-labor intensive traditional crops, such as millet and sorghum [30]. The authors described how unfarmed land reverted to unmanageable plots that were eventually overrun by weedy grasses, leaving behind infertile soil [30].

Another growing concern is the unsustainable harvesting of natural resources. Examples of other unsustainable practices that have been previously documented include uncontrolled charcoal mining, fishing, hunting, and the gathering of protected plants by individuals striving to make a living [31,44,45]. One such example is the gathering and exportation of wild orchids in Tanzania. Wild orchids are considered illegal for trade in Tanzania; however, many of these edible orchids constitute a portion of the diet for neighboring Zambia [31]. This has led to the prohibited trade and exportation of orchids to the Zambian people by Tanzanians, often orphaned youth, whose lives have been affected by HIV/AIDS [31]. Unfortunately, the practice of orchid gathering is not viable, as it often involves uprooting the entire plant, which threatens the survival of many orchid species [31]. Researchers conducted a study to describe these gathering practices in three rural villages of Tanzania [32]. The researchers noted an increase in the prevalence of non-edible orchid species but a decrease in edible orchid species [32]. This shift in orchid type is evidence of ongoing illegal exportation and the endangered existence of edible orchids in this area by individuals who have been affected by this disease [32].

### 5.2. Loss of Human Capacity

The crux of the problem can often be attributed to the loss of human lives, specifically the loss of prime-age adult workers. The World Wildlife Fund, Inc., a non-governmental organization dedicated to environment conservation and restoration efforts, has cited a loss of human capacity due to HIV/AIDS as a major hindrance to preservation efforts [46]. The organization explains that many natural areas cannot be properly protected due to a decrease in highly trained staff, including national park guards and patrol officers [33,46]. This has given way to an increase in unmonitored poaching of endangered species including the buffalo and elephants [46]. Rosen *et al.* conducted a study among Zambian Wildlife Authority officers to assess the influence of AIDS on worker productivity [33]. After adjusting for age and worksite, the disease was associated with a 68% decrease (*p* < 0.0001) in the amount of days on patrol, during the year of observation, compared to the previous year [33]. The investigators also documented an overall decrease in service delivery capacity due to days of absenteeism; days were lost to attend funerals and to replace lost employees [33]. The loss of worker productivity and rising absenteeism is yet another challenge to preserving the relationship between rural life and its impact on the environment.

## 6. Recommendations for Reform

Many of the problems and unique challenges in preserving the environment and social structure of rural sub-Saharan Africa appear to be closely linked to the loss of life. Unfortunately, reversing these deficiencies is a great feat to overcome. Ultimately, HIV/AIDS prevention is imperative to ending the pandemic and relieving the strain the disease places on the environment. Despite the prevalence of disease in sub-Saharan Africa, many of these countries appear to be underrepresented in terms of study location sites, in the identified articles. Strong governmental legislation is ideal for implementing policies geared towards disease prevention, but this may not be feasible for those nations that lack political stability. Still, through education, collaboration, and the active involvement of the population, many actions initiated at the community-level can be helpful to strengthen human capacity and to decrease the disease’s environmental impacts.

Increasing HIV/AIDS awareness and overcoming the stigma of disease in all sectors of rural life is imperative to sparking reform and ultimately attaining prevention. Programs should ensure people understand: (1) what HIV/AIDS is; (2) how it is transmitted; (3) how it affects daily living; and (4) how it can change one’s dependency on natural resources. This knowledge can be disseminated by a variety of methods including, but not limited to, hosting educational sessions, training workshops, community meetings, focus groups, and wellness programs by peer educators and community leaders. As the disease affects all aspects of life, any newly adopted initiatives should be tailored accordingly (*i.e*., for the workplace, places of worship or communal gathering, or for the home).

Initiatives should also be tailored to encompass special programming for populations that are disproportionately affected by HIV/AIDS. For example, females may benefit from learning effective techniques to urge their male partners to create a living will, which explicitly designates the female (or other surviving family members), as the sole land inheritor. They may also benefit from information regarding sustainable alternatives to agricultural livelihood, such as agroforestry and crop species diversification [44,47]. Older adults should be encouraged to transfer traditional wisdom and indigenous practices involving their natural surroundings to the youth in order to preserve cultural knowledge. Programs should also target the youth, as education at a young age may transcend into positive outcomes in the future. Ultimately, all members of the rural community play a role in this effort. Communication, collaboration, and the sharing of ideas and experiences can bolster the shared vision of lessening the impact on the natural environment. Lastly, future research may be warranted in some of the less common focus areas as identified within this review, such as the loss of human capacity and the age dynamic of HIV/AIDS in rural communities.

## 7. Conclusions

HIV/AIDS continues to pose an array of concerns for sub-Saharan Africa. The spread of HIV/AIDS further strains the fragile relationship that has long existed between the local environment, social infrastructure, and rural livelihood. Changing population dynamics and a growing dependency on the environment and its resources are at the center of this crisis. Nevertheless, plausible solutions to overcome some of these problems do exist. If implemented, rural communities of sub-Saharan Africa can effectively work toward environmental preservation.

## Figures and Tables

**Table 1:** Retrieved articles classified by focus area.

Focus Area	Citation	Country	Reference
Rural dimension	Potgieter, N.; *et al*. 2007.	South Africa	[7]
	Lule, J.R.; *et al*. 2005.	Uganda	[8]
	Dorward, A.R.; *et al*. 2006.	Malawi	[5]
	Chapoto, A.; *et al*. 2008.	Zambia	[9]
	Bachmann, M.O.; *et al*. 2003.	South Africa	[10]
	Fagbemissi, R.; *et al*. 2008.	Benin	[11]
Food insecurity	Thangata, P.H.; *et al*. 2007.	Malawi	[12]
	Kaschula, S. 2008.	South Africa	[13]
	Hunter, L.M.; *et al*. 2007.	South Africa	[6]
Rural migration	Mmbaga, E.J.; *et al*. 2008.	Tanzania	[14]
	Clark, S.J.; *et al*. 2007.	South Africa	[15]
	Coffee, M.; *et al*. 2007.	South Africa	[16]
Age dynamic	Schatz, E.; *et al*. 2007.	South Africa	[17]
Gender dynamic	Parker, D.C.; *et al*. 2009.	Uganda	[18]
	Kipp, W.; *et al*. 2007.	Uganda	[19]
	Dunkle, K.L.; *et al*. 2004.	South Africa	[20]
	Kanyamurwa, J.M.; *et al*. 2007.	Uganda	[21]
Unsustainable use of natural resources	Hunter, L.M.; *et al*. 2011.	South Africa	[22]
	Murphy, L. 2008.	Kenya	[23]
	Frank, E.; *et al*. 2008.	Zambia	[24]
	McGarry, D.K.; *et al*. 2009.	South Africa	[25]
	Hunter, S.S.; *et al*. 1993.	Uganda	[26]
	Ebanyat, P.; *et al*. 2010.	Uganda	[27]
	Aliber, M.; *et al*. 2006.	Kenya	[28]
	Drimie, S.; *et al*. 2003.	Kenya, Lesotho, and South Africa	[29]
	Misiko, M.; *et al*. 2008.	Kenya	[30]
	Challe, J.F.; *et al*. 2009.	Tanzania	[31]
	Challe, J.F.X.; *et al*. 2008.	Tanzania	[32]
Loss of human capacity	Rosen, S.; *et al*. 2007.	Zambia	[33]
